# Acute bottlenecks to the survival of juvenile *Pygoscelis* penguins occur immediately after fledging

**DOI:** 10.1098/rsbl.2020.0645

**Published:** 2020-12-16

**Authors:** Jefferson T. Hinke, George M. Watters, Christian S. Reiss, Jarrod A. Santora, M. Mercedes Santos

**Affiliations:** 1 Antarctic Ecosystem Research Division, Southwest Fisheries Science Center, National Marine Fisheries Service, National Oceanic and Atmospheric Administration, La Jolla, CA 92037, USA; 2 Fisheries Ecology Division, Southwest Fisheries Science Center, National Marine Fisheries Service, National Oceanic and Atmospheric Administration, Santa Cruz, CA 95060, USA; 3 Departamento Biología de Predadores Tope, Instituto Antártico Argentino, San Martín B1650CSP, Argentina; 4 Laboratorios Anexos, Facultad de Ciencias Naturales y Museo, Universidad Nacional de La Plata, La Plata B1904AMA, Argentina

**Keywords:** telemetry, Antarctica, recruitment, seabird, *Pygoscelis*

## Abstract

Estimating when and where survival bottlenecks occur in free-ranging marine predators is critical for effective demographic monitoring and spatial planning. This is particularly relevant to juvenile stages of long-lived species for which direct observations of death are typically not possible. We used satellite telemetry data from fledgling Adélie, chinstrap and gentoo penguins near the Antarctic Peninsula to estimate the spatio-temporal scale of a bottleneck after fledging. Fledglings were tracked up to 106 days over distances of up to 2140 km. Cumulative losses of tags increased to 73% within 16 days of deployment, followed by an order-of-magnitude reduction in loss rates thereafter. The timing and location of tag losses were consistent with at-sea observations of penguin carcasses and bioenergetics simulations of mass loss to thresholds associated with low recruitment probability. A bootstrapping procedure is used to assess tag loss owing to death versus other factors. Results suggest insensitivity in the timing of the bottleneck and quantify plausible ranges of mortality rates within the bottleneck. The weight of evidence indicates that a survival bottleneck for fledgling penguins is acute, attributable to predation and starvation, and may account for at least 33% of juvenile mortality.

## 1. Introduction

Ecological theory of top-down and bottom-up effects on recruitment of juveniles to breeding populations predicts that top-down effects (i.e. predation) are likely to be strongest during early life stages when individuals are small or slow growing, while bottom-up effects (i.e. resource limitation) are more important over longer periods [1]. These predictions are particularly relevant for long-lived vertebrates that exhibit high adult survival rates but lower survival during juvenile stages that can extend for multiple years. Survival during such long juvenile stages, however, is likely to vary in space and time, rendering the balance of top-down and bottom-up effects on survival difficult to identify. For example, mark–recapture methods provide age- or stage-specific survival rates but cannot resolve variation in survival between release and initial recapture [2]. Bottlenecks in juvenile survival, however, may be acute before initial recapture, particularly if transitions between rearing and foraging habitats are accompanied by high predation rates [3] or if reduced foraging efficiency of novice predators [4] slows their development or increases starvation risk. Therefore, identifying when and where survival bottlenecks occur for free-ranging marine predators can improve demographic and population modelling and help prioritize effective spatial management efforts [5].

Bottlenecks in juvenile survival may occur among penguins. Once parental care ceases, fledgling penguins transition from terrestrial nesting sites to forage independently in marine habitats where predation risk can be high [6]. The interval between fledging and first return to the natal colony—hereafter the ‘juvenile’ period—can last 1–5 years, depending on the species. Subsequent recruitment to breeding populations is variable but often less than 20% [7], indicative of high mortality during the juvenile stage. However, dispersal of juveniles to distant foraging habitats hinders direct observations of when, where and why death occurs, inhibiting estimation of the spatio-temporal scale of survival bottlenecks.

Satellite telemetry may provide useful data to estimate when and where a survival bottleneck occurs [8,9]. Tags cease transmitting after deployment for myriad reasons [10] but changes in the rate at which tag losses accumulate may help define a bottleneck. We tracked fledgling penguins in the Antarctic Peninsula (AP) region to quantify the spatio-temporal scale of a survival bottleneck and account for factors other than natural death that could lead to tag loss. We corroborate the timing and location of this bottleneck with at-sea observations of penguin carcasses [11], and bioenergetics [12] simulations of mass loss to thresholds associated with low recruitment probability [13]. We address tag shedding and power failures, prominent factors that can lead to tag loss in tracking studies [10], by comparing loss rates from a simultaneous study on adult penguins [14,15], and examining battery voltages reported during deployments.

## 2. Material and methods

We tracked 29 Adélie (*Pygoscelis adeliae*), 8 chinstrap (*P. antarcticus*) and 10 gentoo (*P. papua*) fledglings with Sirtrack Kiwisat-202 K2G-172A satellite transmitters (see electronic supplementary material for details) from four breeding colonies in the AP in 2017 and 2018 (table 1). Fledglings were captured on natal beaches, weighed and transmitters were glued to the dorsal plumage (see electronic supplementary material for details). We tagged fledglings larger than their species-specific historical mean fledgling masses (electronic supplemental material, figure S1) to minimize the potential impacts of tags on fledgling energetics and survival rates.

**Table 1.** Tagging locations, colony sizes, numbers tracked, means and standard deviations of fledgling weights, and medians and ranges (in parentheses) of deployment durations and distances achieved.

species	colony	colony size	longitude	latitude	year	*N*	fledgling weights (g)	duration (d)	distance (km)
Adélie	Admiralty Bay	2200 [16]	−58.446	−62.175	2017	10	3650 ± 210	6.0 (0.2–80.9)	228 (23–1352)
					2018	10	3885 ± 431	7.2 (3.1–52.2)	229 (7–1776)
	Esperanza	104139 [17]	−57.01	−63.4	2018	9	3756 ± 270	9.9 (1.3–106.1)	382 (28–1165)
Chinstrap	Cape Shirreff	2449 [14]	−60.792	−62.46	2017	4	3400 ± 91	9.2 (1.8–50.3)	121 (61–2140)
	Cierva Cove	4846 [18]	−60.984	−64.143	2017	4	3575 ± 506	8.7 (1.7–20.9)	164 (121–223)
Gentoo	Cape Shirreff	705 [16]	−60.792	−62.46	2017	5	4610 ± 345	20 (12.9–75.8)	16 (4–101)
	Cierva Cove	6270 [18]	−60.984	−64.143	2017	5	4840 ± 546	13.2 (9–85.7)	40 (13–72)

Given small sample sizes for each species and site, we pooled data for analyses. We fitted linear and segmented linear models [19] using R [20] to the cumulative proportion of tags lost over time to estimate a single breakpoint, via maximum likelihood, in the rate of tag loss and the magnitude of loss at the breakpoint. We used ANOVA to assess the selection of the segmented models over non-segmented models.

We assess potential effects of technical or physical tag malfunction, failed attachments and tag-induced mortality on observed loss rates with a bootstrap procedure to simulate tag loss for reasons other than natural animal death. We weighted sampling by the inverse of deployment duration to preferentially reject short-duration deployments, assuming that technical and attachment failures occur relatively quickly. We bootstrapped the pooled data 100 times without replacement using rejection rates between 20% and 80%, representing loss rates owing to factors other than death. We fitted segmented models to each sample to estimate the sensitivity of the breakpoint and the magnitude of tag loss attributable to death at the breakpoint. We the addressed effects of attachment failure by comparing loss rates of tags simultaneously deployed on adult penguins at the same sites with the same attachment methods [14,15], expecting similar rates of attachment failure. Finally, we assessed the effects of power failure with battery voltage data. Additional details on bootstrapping and voltage data are in the electronic supplementary material (electronic supplemental material, figures S6–S9).

At-sea sightings of dead penguins, noting that species identification was not possible owing to depredated or scavenged status of carcasses, derive from research cruises [11] conducted between January and March 2003–2011.

We used a bioenergetics model developed for adult Adélie penguins [12] to estimate the time required for body mass to drop below critical thresholds (*M_crit_*) given consumption of 0%, 50% and 90% of maintenance requirements. We used a binomial generalized linear model fitted to the relationship between fledgling mass and recruitment status from mark–recapture data (electronic supplementary material, figure S2) to estimate two values for *M_crit_*, the masses achieving, respectively, 50% (*M_crit50_*) and 10% (*M_crit10_*) of maximum recruitment rates. We used published parameters [12] to run the model, but used the mean mass of Adélie fledglings tracked (3.76 kg) as the initial mass and assumed a 100% chance of being at sea (table 1).

## 3. Results

**Figure 1.**
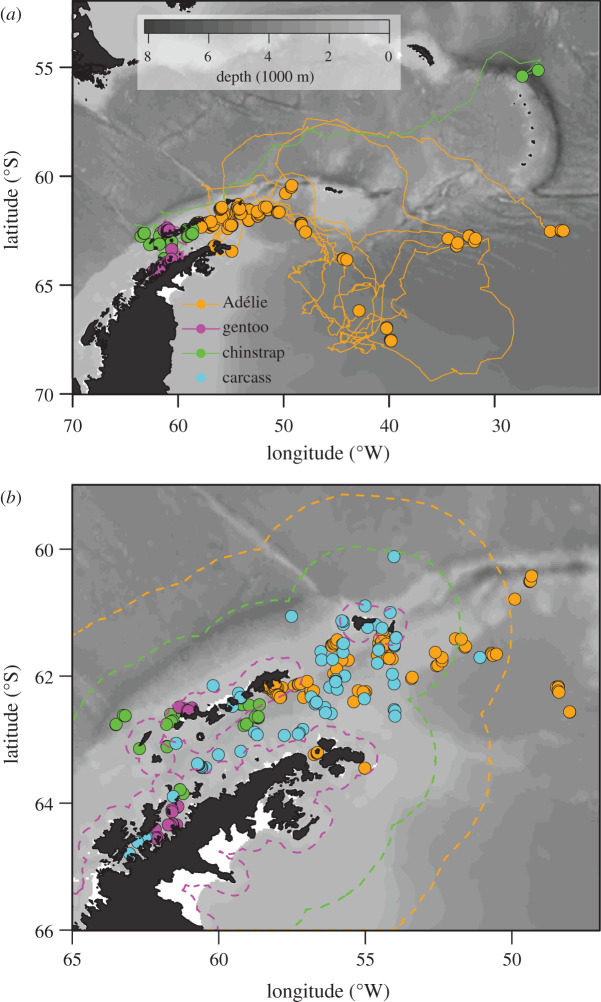
(*a*) Raw tracks (lines) and locations (points) from the last 24 h of each deployment for Adélie, chinstrap and gentoo fledglings. (*b*) Close-up of last-known location estimates and carcass sightings (points). Species-specific median of the distances achieved by each tag within 16 days, plotted as coastal buffers (dashed lines), delineate the spatial scale of the bottleneck.

Fledglings were tracked for up to 106 days covering distances up to 2140 km (table 1). Movements into the Scotia and Weddell Seas were evident for Adélie and chinstrap penguins; gentoo penguins remained coastal (figure 1*a*). Tag loss for all species was concentrated over the continental shelf around the South Shetland Islands and AP (figure 1*b*) and overlapped the spatial (figure 1*b*) and temporal (electronic supplementary material, figure S3) extent of penguin carcasses observed at sea.

**Figure 2.**
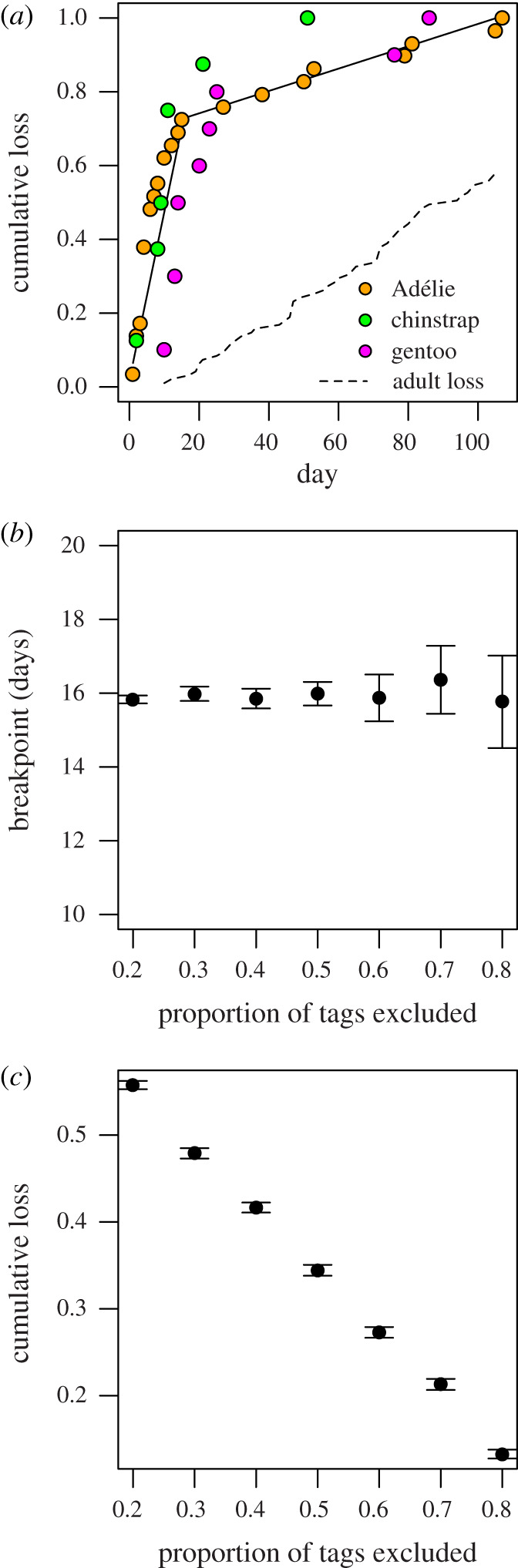
(*a*) Segmented model fit to the pooled cumulative proportion of lost tags (black line). Points indicate species-specific cumulative losses for reference. The dashed line indicates tag loss rates for adults simultaneously tracked from the same colonies [14,15]. Sensitivity of the (*b*) breakpoint estimates and (*c*) cumulative tag losses owing to death at the breakpoint for different tag failure scenarios.

Cumulative proportions of tags lost over time exhibited a segmented relationship (figure 2*a*) with a breakpoint at 15.6 ± 0.76 (95% CI) days, corresponding to 73% tag loss. The mean daily proportion of tags lost before the breakpoint (0.45 ± 0.001; s.e.) was an order of magnitude higher than after (0.003 ± 0.001; s.e.), indicating a bottleneck. Tag loss rates were higher among juveniles than adults that were tagged using the same attachment methods and tracked simultaneously (figure 2*a*). Fledgling loss rates after the breakpoint were similar to adults, suggesting that the bottleneck is acute and specific to fledglings.

Bootstrapping revealed the estimated breakpoint was robust to different data rejection rates (figure 2*b*), but the magnitude of loss attributable to death declined as tag loss increased for other reasons. Failure rates in many tracking studies average roughly 50% [10]. Using this benchmark, the expected loss from death by the breakpoint was estimated at 33%, corresponding to 40% of losses leading to mean recruitment rates of banded cohorts [8] (electronic supplementary material, figure S4).

Species-specific, median distances achieved by the breakpoint (figure 1*b*) provide conservative boundaries of a bottleneck throughout the AP region. Coastal and shelf regions in the northeastern Bransfield Strait and south of Elephant Island were key bottleneck zones for all species (figure 1*b*).

The bioenergetics model predicted that individuals unable to find sufficient food could reach critical masses within timelines similar to the breakpoint in cumulative tag loss (table 2, electronic supplementary material, figures S10 and S11). In the no-ration scenario, *M_crit10_* and *M_crit50_* were reached within 15 days (table 2). The *M_crit10_* was achieved within one month on a 50% ration, while higher rations prolonged mass loss past either level to greater than or equal to 100 days.

**Table 2.** Expected times required for fledglings to reach masses at which they would be expected to recruit at 50% and 10% of the maximum recruitment rate (*M_crit50_* and *M_crit10_*) given daily rations equal to 0%, 50% and 90% of maintenance requirements. Loss owing to death reflects the range of tags lost assuming 50% failure and no failure.

threshold	kg	ration (%)	time (d)	loss owing to death (%)
*M_crit50_*	2.125	0	10	21–48
		50	19	35–74
		90	100	48–98
*M_crit10_*	1.575	0	15	31–70
		50	31	37–77
		90	153	57–100

Battery failure appears unlikely to have affected our results. Voltages at the time of signal loss were normal (P. O'Flaherty 2020, personal communication) and consistent with average voltages reported during each deployment (electronic supplementary material, figure S9).

## 4. Discussion

High attrition rates of tagged fledglings immediately following fledging in near shore and continental shelf regions is consistent with high mortality rates of a survival bottleneck. We examined independent lines of evidence and assumptions about causes of tag failure to infer that an acute bottleneck in fledgling survival is estimable from satellite tracking data.

Several lines of evidence support this inference. First, the rapid loss of tags within 16 days of fledging followed by an order-of-magnitude reduction in loss rates thereafter were robust to bootstrapping scenarios that excluded up to 80% of the data. Second, a recent review reported that most tracking studies experience premature failures in 50% of deployments [10], with battery and attachment failures among the culprits. Voltage data reported by our tags suggest that battery failure, the leading cause of most technical tag failures [10], was unlikely to have contributed to early loss in our study. Moreover, tag loss rates from fledglings and adults immediately after release were markedly different despite using the same tagging methods. Fledgling attachments should have been as robust as adult attachments. Attachment failure, therefore, appears unlikely to cause the rapid loss rate from fledglings. Together, these observations suggest premature failures in our study were likely less than 50%, but this value provides a conservative benchmark to assess mortality in the bottleneck. Simulated failures of 50% estimate that losses owing to mortality could have accounted for 33% of total losses observed within the bottleneck. Such losses, accrued within 16 days of fledging, represent 40% of total losses observed among banded cohorts over the first few years of life [8]. Assuming all tag losses were owing to mortality provides an upper bound on this value; the 73% of tags lost in the bottleneck represents 88% of total losses observed among banded cohorts. The weight of evidence supports the identification of an acute bottleneck in the survival of fledgling penguins, occurring within a few weeks after departure from natal colonies.

Acute mortality among fledglings may arise from predation, insufficient energy acquisition for thermoregulation and growth, or synergies between these factors. While we cannot definitively differentiate the two sources of mortality, our data suggest that predation is an important factor. Most tag loss occurred before critical thresholds relating to low recruitment probability were reached, noting such thresholds do not index imminent death from starvation. Moreover, observations of carcasses at sea and on breeding beaches (electronic supplemental material, figure S5) further support the inference that juvenile penguins are exposed to intense predation pressure after fledging, consistent with top-down effects predicted from ecological theory [1].

Increased predation risk on fledgling penguins may be enhanced by behavioural and developmental limits on resource acquisition. Fledglings are novice swimmers and foragers, receiving little to no support from experienced adults during their transition to independence [21]. Such traits increase vulnerability to predation, especially near natal colonies where predictably high concentrations of prey attract predators. Leopard seal (*Hydrurga leptonyx*), fur seal (*Arctocephalus gazella*) and giant petrel (*Macronectes giganteus*) predation on fledglings near natal colonies is common and can be intense [6,22,23]. Spatially, the bottleneck (figure 1*b*) includes abundance hotspots for fur seals and giant petrels [24] in the Bransfield Strait and areas south of Elephant Island. While avoiding predators in these areas, fledglings must find and capture sufficient food. Relatively poor foraging efficiency by juvenile birds [4] suggests that fledglings may not achieve maintenance rations, thus impeding development. A synergy between potentially limited resource acquisition, slow growth and high predation pressure near natal colonies provides a plausible mechanism for an acute survival bottleneck. Note that animals with a lower condition would approach critical thresholds more rapidly than those we tracked (electronic supplementary material, figures S10 and S11). If mortality rates owing to starvation or predation increase with reduced body condition, mortality within the bottleneck may be higher than estimated for the relatively larger individuals tracked in our study.

A survival bottleneck for fledgling penguins occurs within three weeks near breeding colonies and over the adjacent continental shelves. Such bottlenecks identify high-priority areas for spatial management efforts, particularly related to the mitigation of fisheries and other human impacts [16,25,26] on penguins. Our conclusion is based on a novel view of tracking ‘failures’ and corroborated with auxiliary datasets to provide insight on a cryptic population process for a group of iconic marine predators. While assumptions about the magnitude of mortality within the bottleneck are unavoidable, our results robustly quantify the spatio-temporal scale of a survival bottleneck and plausible ranges of mortality that represent a large proportion of total losses accrued over the juvenile period.

## Supplementary Material

Supplementary Methods and Results

## Data accessibility

Data for this article are available in the Dryad Digital Repository at: https://dx.doi.org/10.5061/dryad.2v6wwpzkh [27].
