# Supplementary material for: Acute bottlenecks to the survival of juvenile Pygoscelis penguins occur immediately after fledging
Source: Biol Lett. 2020 Dec 16;16(12):20200645. doi: 10.1098/rsbl.2020.0645 (PMC7775978; doi:10.1098/rsbl.2020.0645)
Supplement: Supplementary Methods and Results [file rsbl20200645supp1.pdf]

Electronic supplementary material for:

Hinke JT, Watters GM, Reiss, CS, Santora JA, Santos MM. 2020 Acute bottlenecks to the survival of juvenile Pygoscelis penguins occur immediately after fledging. *Biol. Lett.* 20200645. (doi: 10.1098/rsbl.2020.0645)

Supplementary Methods and Results

Data availability

All data used in this supplementary material are publically available [1].

Tag information and tagging procedures

We used Sirtrak Kiwisat K2G-172A ARGOS telemetry tags for this study. The tags have dimensions of 60 x 27 x 17mm, weigh 32g, and are depth rated to 250m. The tags were pre-programmed by the manufacturer to attempt location estimation daily between 12:00 and 18:00 UTC. As programmed, expected life of the battery was 6 months.

Tag weight represented <1.07% of all fledgling weights, adhering to recommendations for birds that transmitter weights be less than 3-5% of body weight [2]. Additionally, the tags were equipped with an 18mm, flexible external antenna, mounted at a 45° angle. The tag was oriented on the animal so that the antenna pointed caudally for all deployments, consistent with best-practice advice for reducing drag on swimming penguins [3].

Tags were mounted directly to the back plumage in a caudal position [4] with cyanoacrylate glue. Two small (2.5x150mm) black plastic cable ties were threaded through underlying contour feathers and closed over the top of the tag as an additional fastener. Small beads of glue were traced along the cable ties on top of the transmitter to help secure the attachment.

Fledgling mass

Fledglings were weighed during standardized periods at Cape Shirreff and in Admiralty Bay when Adélie and chinstrap penguins move from their nesting sites to seaside beaches just prior to departure. Chicks were caught on the beaches at that time and weighed with spring scales. Gentoo penguin chicks do not depart en masse, so we used a standard age of 85 days (measured from the median egg lay date in the colony) to estimate their fledgling weight. We used the mean weights from all years of data collection which comprises 5833 Adélie fledglings from Admiralty Bay colonies over 34 years between 1982 and 2019, 4942 chinstrap fledglings

from Cape Shirreff over 23 years between 1997 and 2019 and 3427 gentoo fledglings from Cape Shirreff over 21 years between 1998 and 2019.

Figure S1. Distribution of historical fledgling weights for Adélie (Admiralty Bay), gentoo, and chinstrap penguins (Cape Shirreff). Vertical dashed lines represent mean mass of fledglings tracked with satellite transmitters.

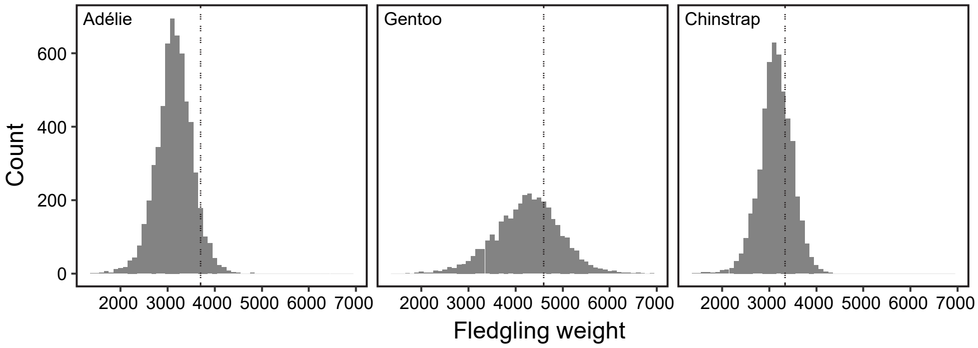

Critical weights and recruitment

We used data from fledgling Adélie penguins that were banded and weighed in Admiralty Bay between 1981 and 2010. Methods of banding have been previously described [5]. In total, 3909 banded and weighed Adélie fledglings were released. The first observation of a banded bird within its natal colony in a subsequent year indicated a successful recruitment event. In total, 11.4% (446) of released birds recruited.

We fitted binomial generalized linear models with linear and quadratic terms to estimate the relationship between weight at fledging and eventual recruitment to the natal colony. We used AIC for model selection, which indicated a quadratic model provided the best fit to the data (figure S2).

With the best fitting model, we estimated critical masses (respectively M_{crit50} and M_{crit10}) at which the probability of recruitment was expected to be 50% and 10% of its maximum level (0.12, figure S2). We estimated critical masses only for the left tail of the quadratic curve because, regardless of initial mass, fledglings would only be expected to lose weight if maintenance rations were not consumed. M_{crit50} was estimated to be 2125 g, and M_{crit10} was estimated to be 1575 g. Note that the smallest fledgling ever observed to subsequently recruit to the breeding population weighed 2100g.

Figure S2. Best-fitting quadratic relationship between fledging weight and recruitment for Adélie penguins in Admiralty Bay. Critical fledgling masses with probabilities of recruiting at 10% (1575 g) and 50% (2125 g) of the maximum recruitment probability are highlighted with red dots. The upper and lower hash marks identify, respectively, the weights of recruited and non-recruited fledglings.

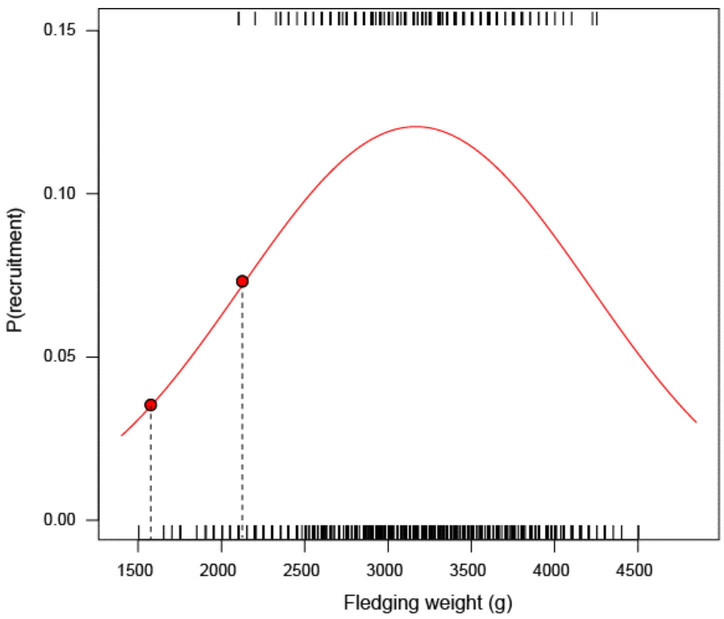

At-sea observations of dead penguins and the timing of fledging

Nine years (2003 – 2011) of at-sea surveys, consisting of two legs that each repeated the same survey grid [6], were conducted throughout January and again in February/March. Observers recorded the locations and numbers of penguin carcasses floating on the sea surface during normal marine mammal and seabird observation periods using methods previously described [7]. Here, we illustrate the distribution of dates when carcasses were observed (figure S3).

The carcass observations correspond to the main fledging periods of Adélie and chinstrap penguins. At independence, Adélie and chinstrap fledglings depart their natal colonies en masse over the course of a few days. The dates for this departure period were recorded at all Admiralty Bay and Cape Shirreff colonies from the time the first fledgling birds are observed on beaches (i.e., no longer associated with their parents or nesting sites) until no more fledglings are found in the colony (i.e., all departed to sea). The timing of this departure period can vary by species, colony location, and year. In our study colonies, these periods typically occur from mid-January to mid-February for Adélie penguins and from late February into March for chinstrap penguins (figure S3). Note that there is no corresponding fledging period for gentoo penguins, since this species does not exhibit mass departures from natal colonies [8].

However, tracked fledgling gentoo penguins generally initiated dispersal by late February or early March [8].

Figure S3. Histogram of the timing of penguin carcass observations from U.S. AMLR cruises, 2003-2011. Overlaid are the historical ranges of fledging dates for Adélie penguins and chinstrap penguins from the Admiralty Bay and Cape Shirreff colonies, as indicated by horizontal lines with end caps. The final dates of location estimates from satellite telemetry for all deployments are indicated with colored dots.

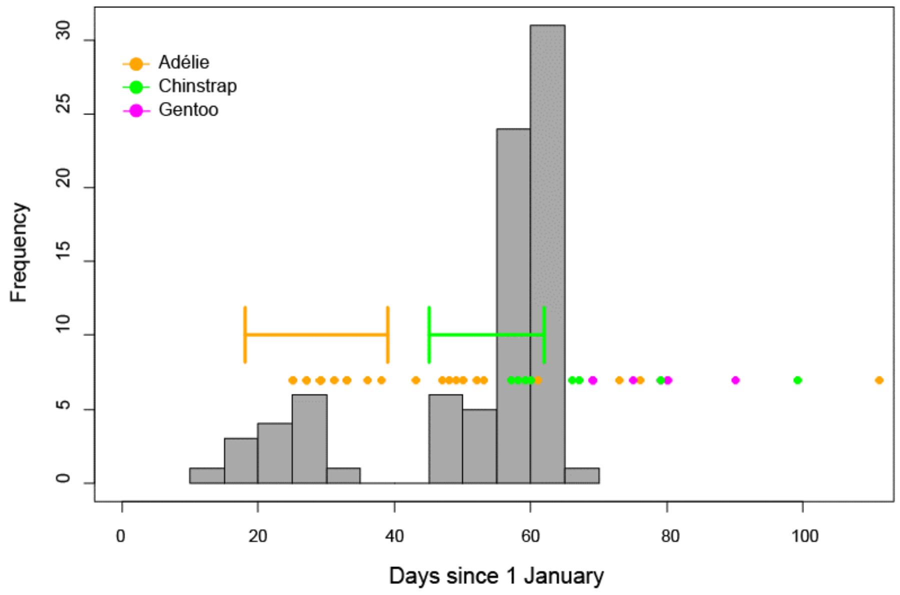

Recruitment indices

Recruitment indices, calculated as the proportion of flipper-banded birds that return to natal colonies and adjusted for likely band-loss rates, have been described previously [5]. We update them here for Adélie, chinstrap, and gentoo penguins from Admiralty Bay and Cape Shirreff colonies. Across species and sites, recruitment rates were variable but averaged 0.20 ± 0.02 for cohorts fledged from 1981 through 2016. This corresponds to an average loss of 80% of banded individuals from a given cohort over the first few years of life.

Figure S4. Updated indices of recruitment for Adélie, chinstrap, and gentoo penguins for cohorts fledged from 1981 through 2016. The average recruitment across all species and sites (20%) is indicated as the dashed horizontal line.

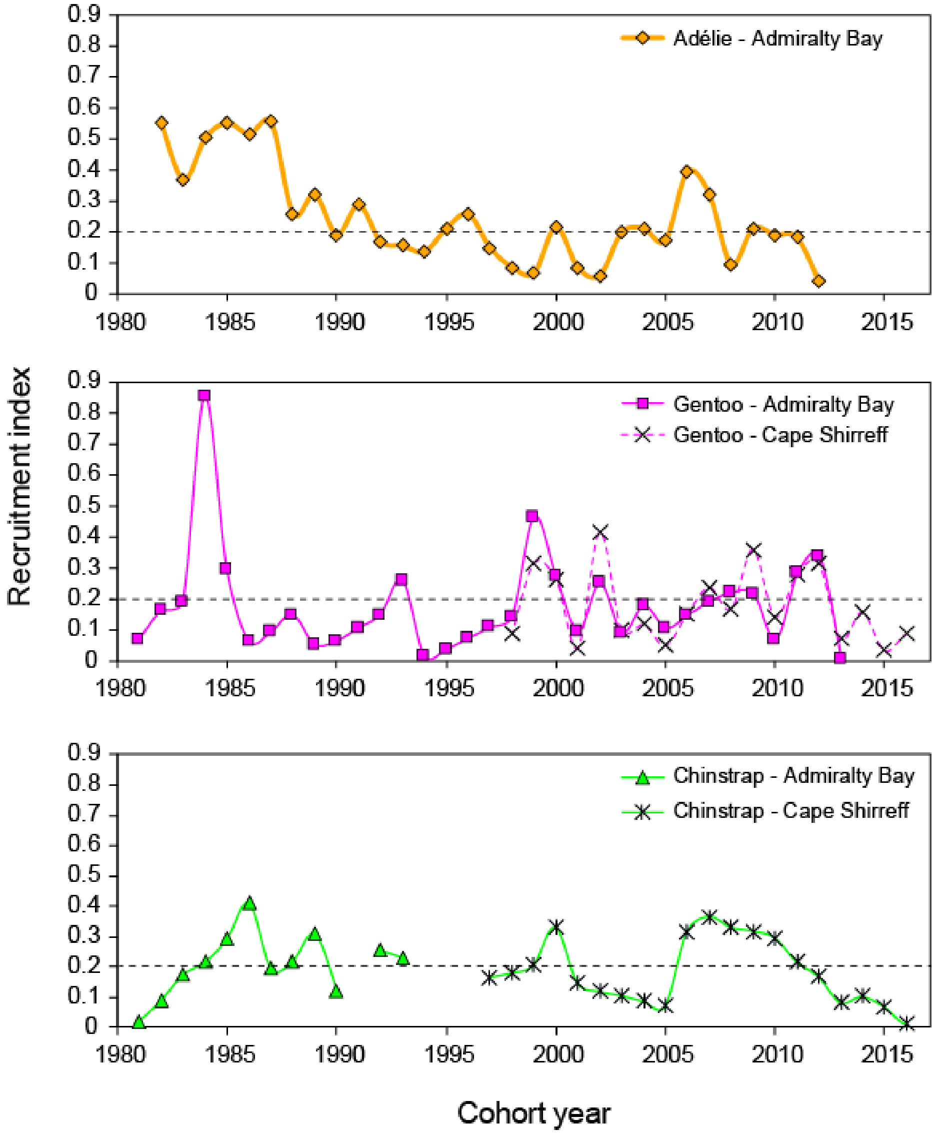

Carcass counts at Cape Shirreff

A count of beach-cast fledgling carcasses is conducted annually at Cape Shirreff. One week after the fledging period for chinstrap penguins ends all accessible beaches are searched for freshly depredated chick carcasses. The data represent a crude and conservative index of local predation, noting that 1) leopard seals, fur seals, and giant petrels are present in the colonies at this time and 2) depredation at sea does not guarantee a carcass will wash ashore. The carcass counts exhibit marked inter-annual variation, with counts exceeding 5% of local chick production in some years.

Figure S5. Annual counts of depredated chinstrap penguin chicks from Cape Shirreff, given as a proportion of annual chinstrap chick production in the colony.

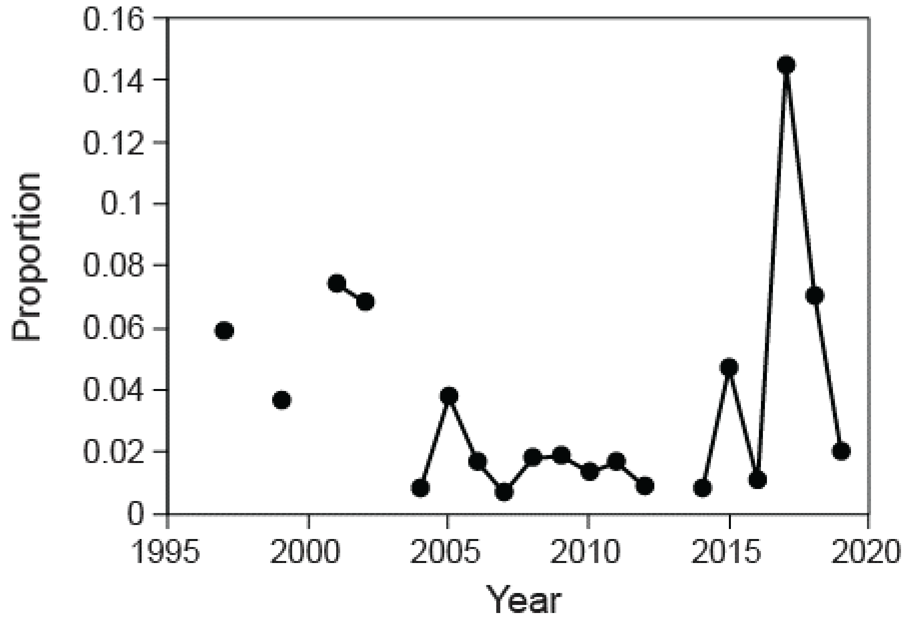

Bootstrapping

We developed a bootstrapping procedure to simulate alternative scenarios of tag failure that may affect the timing of a survival bottleneck and magnitude of mortality estimated within the bottleneck. Specifically, we evaluate how the timing of the bottleneck and the magnitude of loss due to animal death varies as increasingly larger proportions of tags are assumed to have been lost for reasons other than animal death.

The bootstrapping algorithm assumes that faulty tags, weak tag attachments, and tags that fail prematurely for other reasons are more likely to fail early, causing transmission of location information to cease. The simulated failed deployments are therefore removed from the bootstrap analysis, leaving only samples that failed due to death.

To simulate such failures, we weighted sampling of the pooled data based on the inverse of deployment duration to reject short-duration deployments preferentially. We sample from the data without replacement to reinforce preferential removal of short-duration deployments. The resulting sample of data represents the tags whose losses are attributable to death, but with mortality estimates that account for the proportion of tags lost due to other reasons.

Note that we tested alternatives to the above, namely by bootstrapping with replacement and by weighting sampling to preferentially reject long-duration deployments. Ultimately, we believe that the methods described above (and in the main paper) provide a conservative approach to quantifying plausible ranges of mortality inside the bottleneck. For example, a weighting that preferentially rejected long-duration deployments effectively increased the magnitude of mortality within the bottleneck but did not affect the timing of the bottleneck.

We rely on a published report [9] that suggests 50% failure rates are common in most tracking studies to establish a plausible lower bound on our estimates of mortality within the bottleneck, though we present results for simulated failure rates from 2% to 80%.

The bootstrapping results for a range of plausible data-rejection rates are provided in figure 2b and figure S6. As more data are rejected due to premature tag failure, the proportion of tags potentially lost to animal death is reduced, but the breakpoint is consistently around 16 days.

Figure S6. Bootstrap simulation results for seven alternative data exclusion rates. Individual bootstrapped samples are plotted in light blue. For visual reference, an overall segmented model fitted to the pooled bootstrap data is in black. The dashed vertical line denotes its breakpoint.

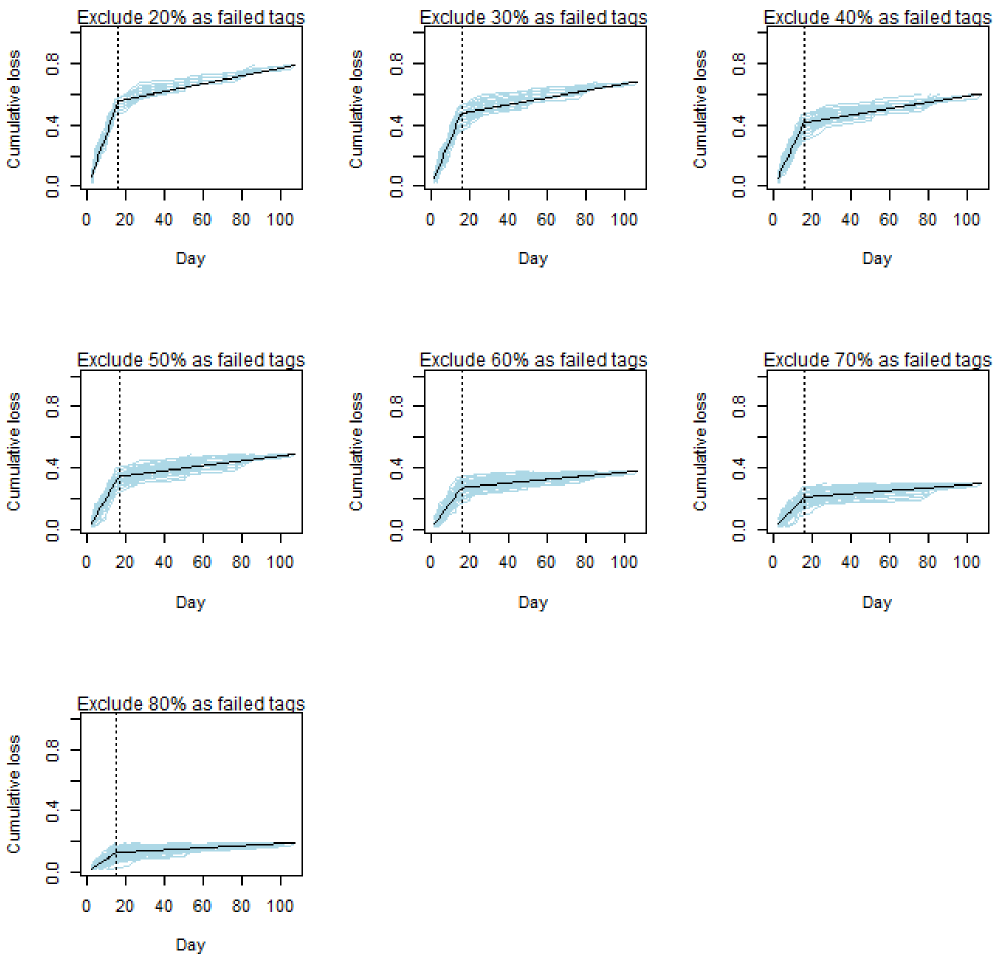

We used ANOVA to assess parsimony of segmented versus non-segmented linear models because the non-segmented linear model is nested within the segmented linear model. We plotted histograms of the p-values from these tests (figure S7). Except for bootstrap samples with the highest rejection rate, segmented models fitted the data better than non-segmented models in all cases. At the highest rejection rate we tested, segmented models fitted the data better than non-segmented models 89% of the time. Note that an 80% rejection rate selects only nine tags for subsequent analysis.

Figure S7. Histogram of p-values from ANOVA of the segmented and non-segmented linear models for each iteration of the bootstrapping procedure for each exclusion rate. Bins are set at a width of 0.01.

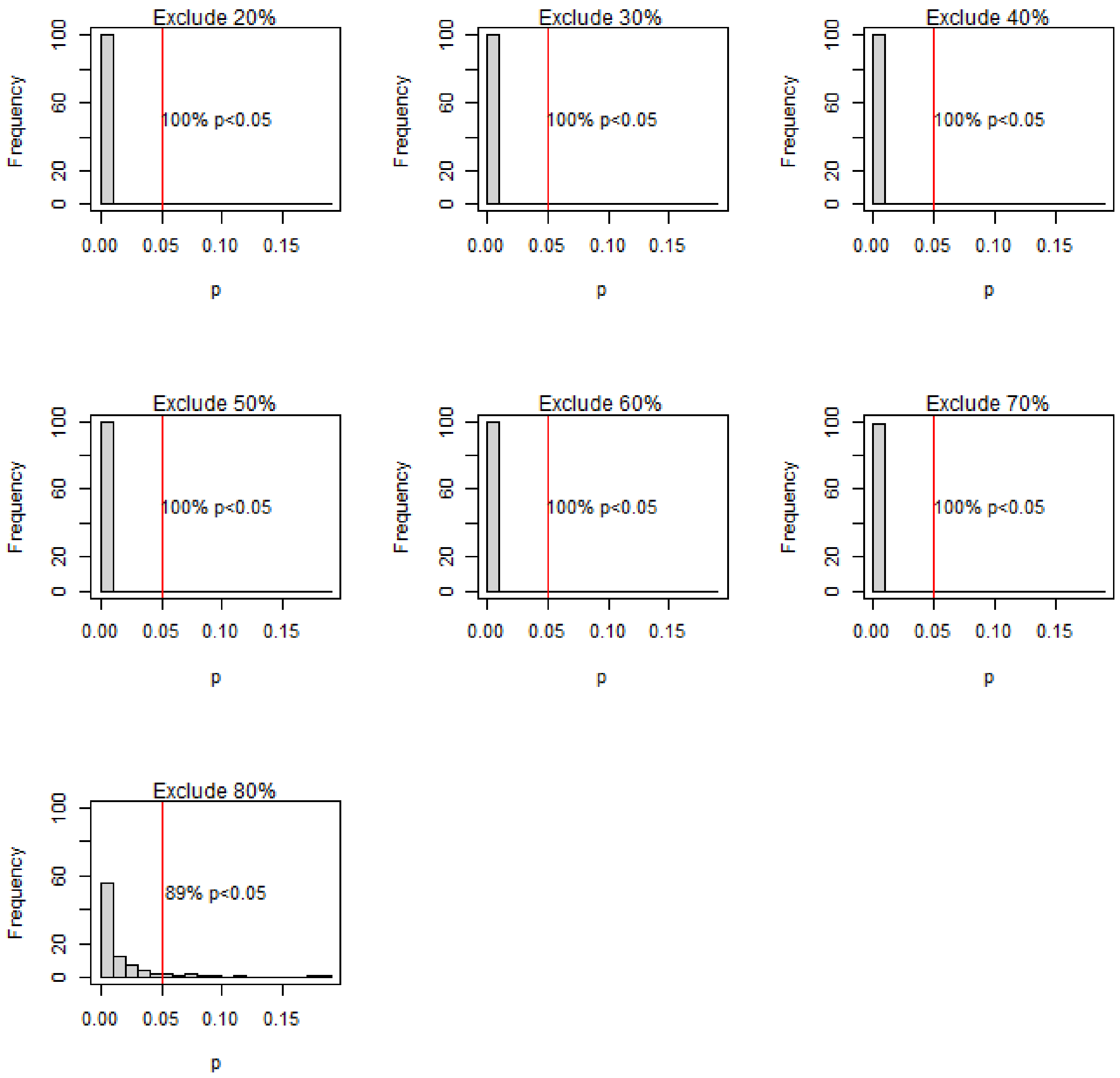

We also computed the ratio of estimated slopes before and after (Beta1 and Beta2 respectively) the estimated breakpoints in our bootstrap samples. In all cases, Beta1 is greater than Beta2, often by an order-of-magnitude. This demonstrates that high loss rates immediately after fledging are insensitive to the proportions of tags lost due to reasons other than animal death.

Figure S8. Histograms of the logged ratio between slope parameters (Beta1 and Beta2) in the segmented models. Values ≥1 indicate order-of-magnitude differences between slopes before and after estimated breakpoints.

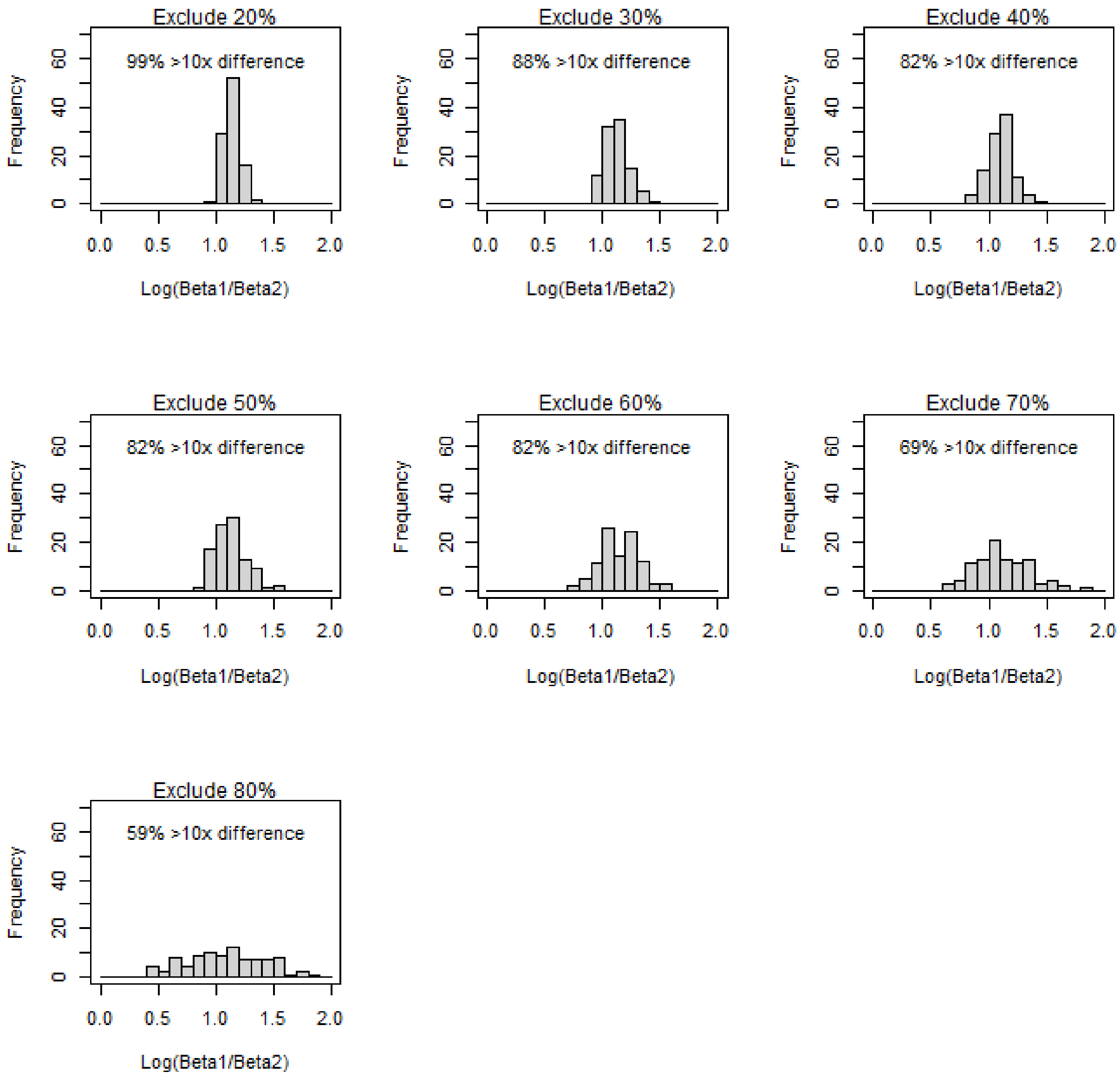

Tag power

Failure of telemetry instruments can arise from technical and mechanical failure of the tag, tag shedding or removal by the animal, and premature animal death. Power failure associated with bad batteries is the number one cause of failure, accounting for roughly 50% of all technical failures [9].

We examined the battery voltages of tags used in this study at the time of their last transmission and over the duration of the deployment prior to each tag's last transmission to assess the likelihood that battery failure was a factor in the rate of tag loss. We find this to be unlikely.

Data were available for the tags released on Adélie penguins in 2018 (main manuscript Table 1). Note that this subset of tags was manufactured at the same time as all tags released in 2017, but these were shelved for one full year due to logistical problems that prevented their deployment until 2018. This shelving might be expected to increase the likelihood of power failure in these 29 tags. However, battery voltages at the time of last transmission remained in "normal" operating ranges for these tags (P. O'Flaherty, pers. comm. 2020). A significant linear trend ($F_{1,17}$=9.7, p=0.006) in the relationship between voltage and duration (figure S9) indicates that tags lost early in their deployment presumably had sufficient power for future transmissions. Moreover, final battery voltages were within a narrow range of values that were comparable to the mean transmission voltages of these batteries. A paired t-test indicates no difference between mean and final transmission voltages (t_{18}=1.47, p=0.16), suggesting that there was no sudden drop-off in voltage at the time of the last transmission. Together, these results suggest that battery failure was not the cause of tag failure in this study.

Figure S9. Relationship between (a) final battery voltage and deployment duration (regression line is included for reference) and (b) mean battery voltage and final battery voltage (1:1 line included for reference).

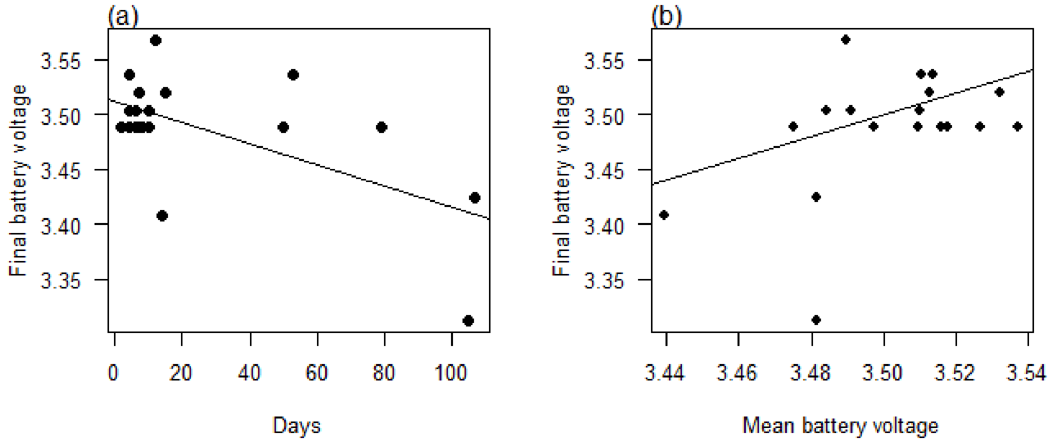

Bioenergetics, body condition, and tag effects

Body condition and increased energy expenditures owing to tagging effects may affect subsequent survival within a post-fledging bottleneck. To assess likely impacts of these two factors on our results, we used the bioenergetics model [10] described previously and used in the main manuscript. We note that the bioenergetics model [10] was developed originally for breeding, adult Adélie penguins, but is here applied to simulate recently fledged penguins. Ontogenetic changes in metabolic rates may cause fledgling energetics to differ from those of adults, but we proceed with published parameterizations [10] given a lack of fledgling-specific parameter estimates at this time. Here, we simulated three no-ration scenarios and three half-ration scenarios to compare mass loss of birds with different initial weights. We repeated the simulations for a range of multipliers for energy expenditure that may arise from the additional drag generated by an externally attached tag [4]. Specifically, we tested 10% and 30% increases in energy expenditure to assess sensitivity of weight loss during the bottleneck period, noting that these multipliers bracket an up-to 20% increase in tag-induced swimming expenditures reported [11] for Magellanic penguins (*Spheniscus magellanicus*).

We used initial weights estimated as: 1) the mean mass of our tagged Adélie penguins (3.76 kg) to address our study results; 2) the historical mean mass of fledglings at our study site (3.16 kg) to assess an average individual; and 3) the lower 25th quantile of fledgling mass at our study site (2.9 kg) to assess a poor-condition fledgling.

Given no rations, fledgling birds at each initial mass are predicted to reach levels associated with low recruitment within the bottleneck period (vertical dashed line in figure S10), with the smallest birds eating no rations achieving the lowest critical mass in 10 days. Half-rations extend the period to attaining critical mass threshold by 10 to 17 days depending on initial mass.

Figure S10. Mass loss over time assuming no rations (dashed lines) or half rations (solid lines) for three different initial masses. The blue lines represent simulations presented in the main text.

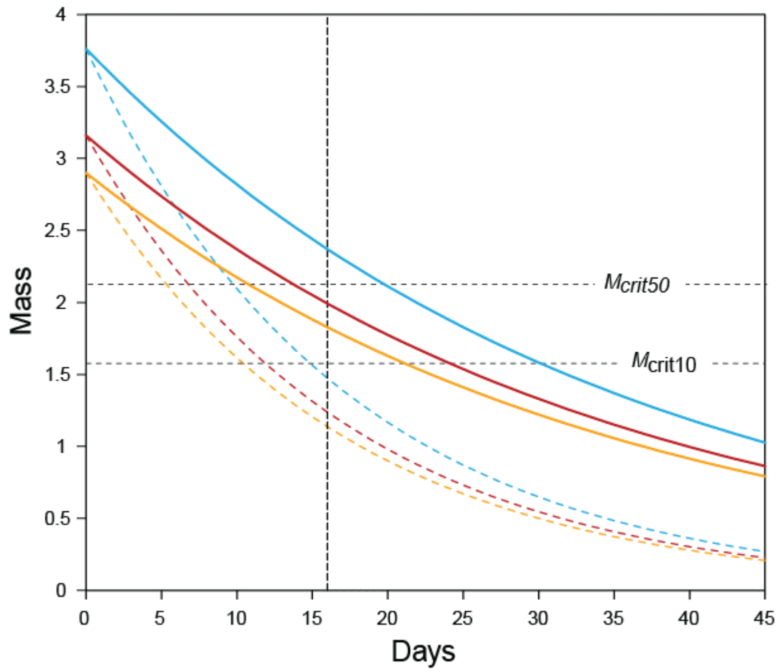

Simulations of mass over time were sensitive to increases in tag-induced energy expenditure, with more rapid loss predicted with increasing effects of tags on necessary energy expenditures (figure S11). The results suggest that no-ration birds reach critical masses 0-1 day sooner at 10% increases in expenditures, and 1-2 days sooner at 30% increases in expenditure. Half-ration birds reach critical masses 1-2 days sooner at 10% increases and up to a week earlier at 30% increases in expenditures. Generally, the results suggest that birds less than average size on rations <50% are likely to reach critical thresholds within the bottleneck period.

Figure S11. Simulation results for three different initial masses (M_o) that assume either no-ration or half-ration and three multipliers for effects of tags on energy expenditures. Vertical solid line marks the 16-day breakpoint identifying the bottleneck. Horizontal dashed and dotted lines mark the critical weights identified in the main manuscript.

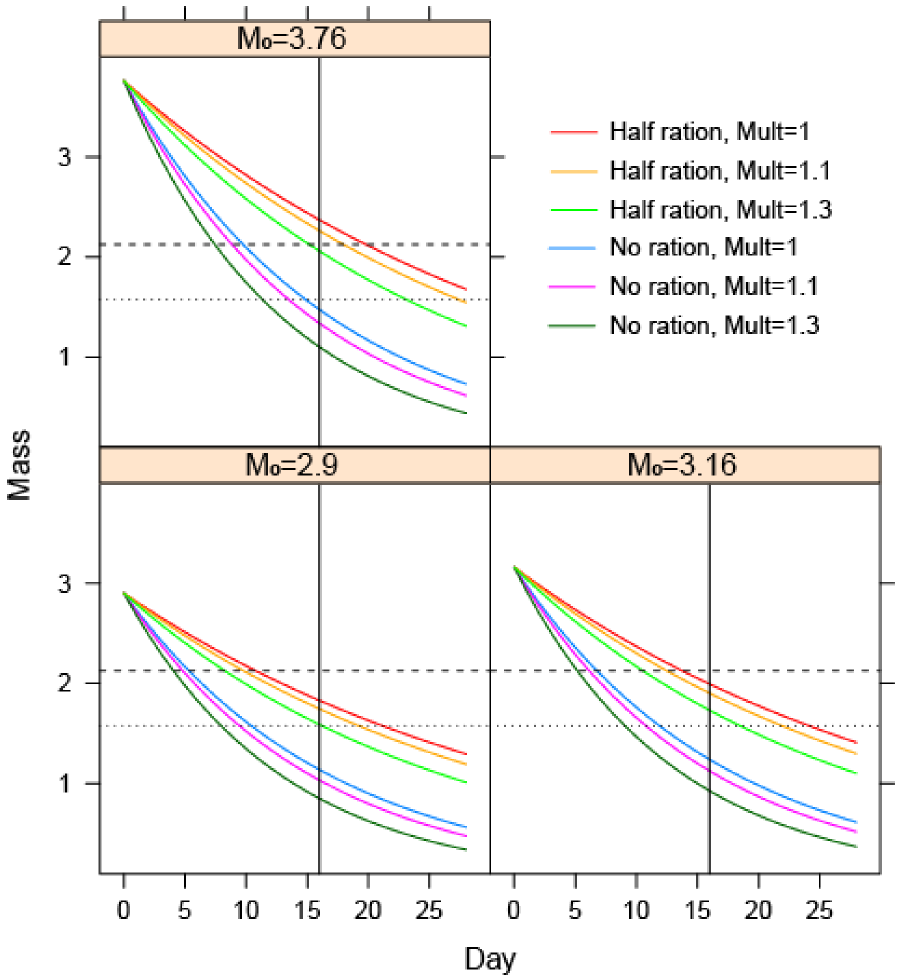

We interpret these simulations to indicate that carrying a tag increases the likelihood of mass loss that would lead to starvation or increased predation risk within the bottleneck period, particularly for small individuals that do not feed.

Finally, historical data demonstrate that fledglings were of average (chinstraps) or above-average (gentoo and Adélie) condition in our study (figure S12).

Figure S12. Mean fledgling mass over time for Adélie penguins at Admiralty Bay, and for gentoo and chinstrap penguins at Cape Shirreff Livingston Islands. The year 2017 is marked with a vertical dashed line. Historical, species-specific mean weights are indicated by dotted horizontal lines.

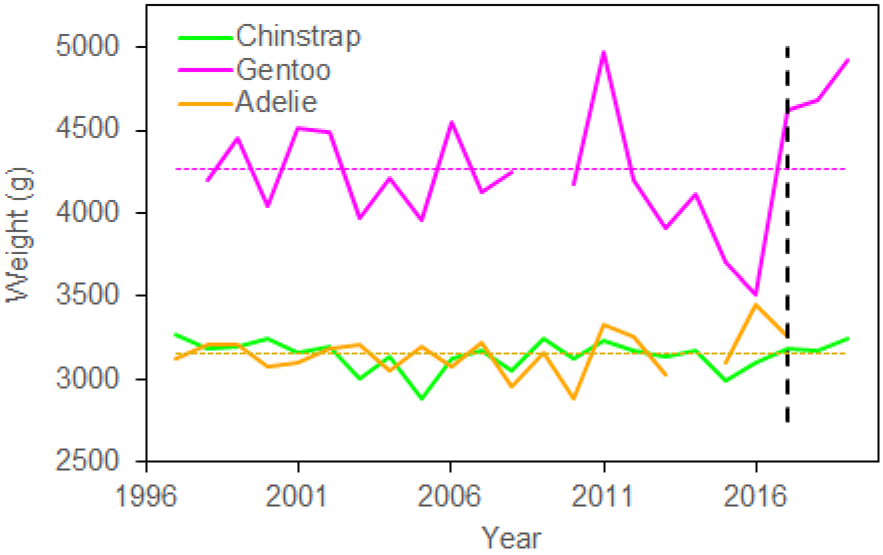

References

1. Hinke JT, Watters GM, Reiss CS, Santora JA, Santos MM. 2020 Data sets supporting "Acute bottlenecks to the survival of juvenile Pygoscelis penguins occur immediately after fledging". *Dryad Digital Repository*. (doi: 10.5061/dryad.2v6wwpzkh)
2. Kenward RE. 2001 A Manual for Wildlife Radio Tagging. Academic Press. London.
3. Wilson RP, Kreye JM, Lucke K, Urquhart H. 2004 Antennae on transmitters on penguins: balancing energy budgets on the high wire. *J Exp Biol.* **207**:2649–62.
4. Bannasch R, Wilson RP, Culik B. 1994 Hydrodynamic aspects of design and attachment of a back-mounted device in penguins. *J. Exp. Biol.* **194**:83-96.
5. Hinke JT, Salwicka K, Trivelpiece SG, Watters GM, Trivelpiece WZ. 2007 Divergent responses of *Pygoscelis* penguins reveal a common environmental driver. *Oecologia* **153**:845-855. (doi: 10.1007/s00442-007-0781-4)
6. Reiss CS, Cossio AM, Loeb V, Demer D. 2008 Variations in the biomass of Antarctic krill (*Euphausia superba*) around the South Shetland Islands, 1996-2006. *ICES J. Mar. Sci.* **65**:497-508. (doi: 10.1093/icesjms/fsn033)
7. Santora JA, Viet RR, Reiss CS, Schroeder ID, Mangel M. 2017 Ecosystem oceanography of seabird hotspots: environmental determinants and relationships with Antarctic krill within an important fishing ground. *Ecosystems* **20**:885-903. (doi: 10.1007/s10021-016-0078-8)

8. Polito MJ, Trivelpiece WZ. 2008 Transition to independence and evidence of extended parental care in the gentoo penguin (*Pygoscelis papua*). *Mar. Biol.* **154**:231-240 (doi: 10.1007/s00227-008-0919-x)
9. Hofman MPG, Hayward MW, Heim M, Marchand P, Rolandsen CM, Mattisson J, Urbano F, Heurich M, Mysterud A, Melzheimer J, et al. 2019 Right on track? Performance of satellite telemetry in terrestrial wildlife research. *PLoS One* **14**: e0216223. (doi: 10.1371/journal.pone.0216223)
10. Southwell D, Emmerson L, Forcada J, Southwell C. 2015 A bioenergetics model for estimating prey consumption by an Adélie penguin population in East Antarctica. *Mar. Ecol. Prog. Ser.* **526:**183-197. (doi: 10.3354/meps11182)
11. Wilson RP, Sala JE, Gómez-Laich A, Cianco J, Quintana F. 2015 Pushed to the limit: food abundance determines tag-induced harm in penguins. *Anim. Welfare* **24**:37-44.
